# Regulation of photosensitisation processes by an RNA aptamer

**DOI:** 10.1038/srep43272

**Published:** 2017-02-24

**Authors:** Tran Thi Thanh Thoa, Noriko Minagawa, Toshiro Aigaki, Yoshihiro Ito, Takanori Uzawa

**Affiliations:** 1 Nano Medical Engineering Laboratory, RIKEN, Wako, Saitama, Japan; 2 Department of Biological Science, Graduate School of Science and Engineering, Tokyo Metropolitan University, Hachioji, Tokyo, Japan; 3 Center for Molecular Biology, Duy Tan University, P402, K7/25 Quang Trung, Danang, Vietnam; 4 Emergent Bioengineering Materials Research Team, RIKEN Center for Emergent Matter Science, Wako, Saitama, Japan

## Abstract

One of the most powerful attributes of proteins is their ability to bind to and modulate the chemistry of cofactors and prosthetic groups. Here, we demonstrated the ability of an artificial nucleic acid (an aptamer) to similarly control the functionality of a non-biological element. Specifically, we selected an RNA aptamer that binds tris(bipyridine) ruthenium (II), Ru(bpy)_3_^2+^, an inorganic complex that has attracted intense interest due to its photoredox chemistry, including its ability to split water by visible light. We found that a newly discovered aptamer strongly and enantioselectively binds Λ-Ru(bpy)_3_^2+^ (*K*_d_ = 65 nM) and, in doing so, selectively suppresses deactivation via energy transfer, thereby elongating the lifetime of its photo-excited state by four-fold. The ability of the aptamer to enhance this important aspect of Ru(bpy)_3_^2+^ chemistry illustrates a broader point concerning the potential power of combining *in vitro*-created biomolecules with non-biological reactants to perform enhanced chemical reactions.

Furnishing coordinates on a transition metal creates various features. Exploring the combination of transition metals and coordinates has satisfied demands for creating an artificial, functional molecule. For example, tris(bipyridine) ruthenium (II) [Ru(bpy)_3_^2+^] has attracted intense interest due to its photoredox chemistry, including its ability to split water into H_2_ and O_2_ by visible light^1^,^2^,^3^,^4^ and singlet-oxygen production for biological applications^5^, as well as its applications in organic synthesis^6^. Ultrafast intersystem crossing from the excited singlet state efficiently produces a functionally important triplet state [the ^3^metal-to-ligand charge transfer (^3^MLCT) state] that possesses a powerful photosensitisation ability for non-mutually exclusive processes: energy- and charge-transfer processes (Fig. 1)^7^,^8^,^9^. Although selective energy- or charge-transfer could play an important role in the control of photochemical reactions, such processes are difficult to regulate.

In this study, we attempted to mimic the natural evolution of protein-based enzymes to furnish a transition-metal complex. Protein-based enzymes modulate, control, and regulate the chemistry of cofactors, such as organic or organometallic prosthetic groups. Various derivatives of prosthetic groups and their surrounding biomolecules (proteins and oligonucleotides) have been investigated and synthesised to tune or mimic the function(s) of protein-based enzymes. However, these strategies are limited to the exploration of similar structures of natural prosthetic groups that fit an existing protein-based enzyme.

Here, we artificially evolved ribonucleic acids to create a non-natural ruthenium complex as a new cofactor. Specifically, we selected an RNA aptamer. Aptamers are typically selected from random oligonucleotide pools by a screening process called Systematic Evolution of Ligands by EXponential enrichment (SELEX)^10^,^11^. The SELEX process provides a means of discovering those rare oligonucleotide sequences that strongly interact with any given specific target. To date, aptamers that have been created recognise a wide variety of targets, including metal ions, low molecular weight organic compounds, proteins, and even whole cells^12^,^13^,^14^,^15^,^16^. Such robustness as to the target molecule has opened a possibility to create uniquely designed binding oligonucleotides to meet specific demands, including drug development, gene regulation, and biosensor development^12^,^13^,^17^,^18^. Here, using the SELEX technique, we selected an aptamer that binds to ruthenium complex and explored its ability to regulate photosensitisation processes.

## Results and Discussion

### *In vitro* selection of a Ru(bpy)_3_^2+^-binding aptamer

**Figure 2:**
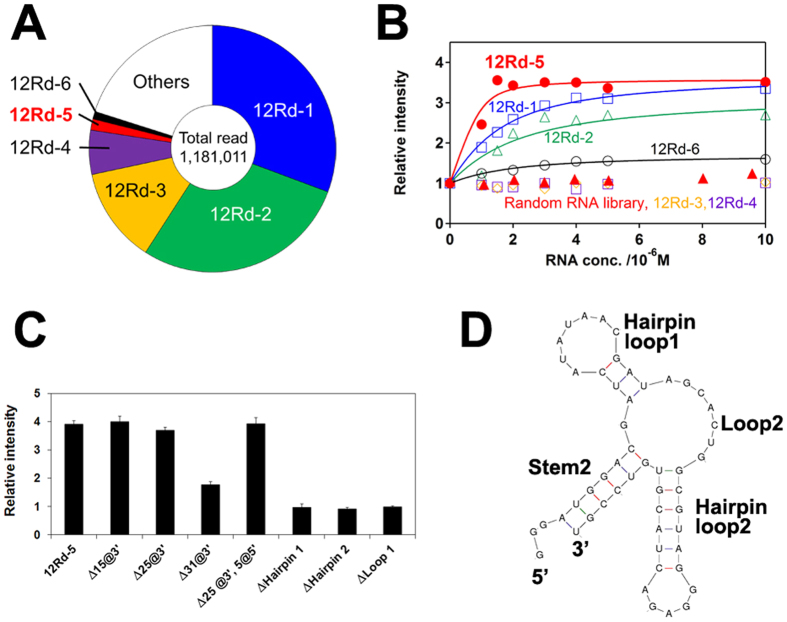
Selection of the RNA aptamer with the highest affinity for Ru(bpy)_3_^2+^. (**A**) The top six selected sequences cover >80% of the total read. (**B**) Titration experiments clarified that the RNA aptamer 12Rd-5 exhibited the highest affinity for Ru(bpy)_3_^2+^. Although 12Rd-1 and 12Rd-2 were well populated after 12 rounds of selection, their affinities for Ru(bpy)_3_^2+^ (*K*_d_ ≈ 1 μM) were lower than that observed for 12Rd-5. (**C**) The titration assay using the truncated 12Rd-5 sequences (Table 1) indicated that the 25-nt sequence at the 3′ end and the 5-nt sequence at the 5′ end (except for the GGG sequence) were not required for Ru(bpy)_3_^2+^ binding, while the other loops and hairpins were required. (**D**) The predicted structure of the Ru(bpy)_3_^2+^-binding aptamer.

We selected several candidates of Ru(bpy)_3_^2+^-binding aptamers by SELEX^10^,^11^. Briefly, we incubated 10^15^ RNA sequences (on average ~100 copies for each sequence) with magnetic beads that were coupled with a Ru(bpy)_3_^2+^ derivative and then washed out the unbound RNA. The bound RNA was enriched by RT-PCR as dsDNA for the next round of selection. After the 12^th^ round of selection, we first cloned the 8^th^ and 12^th^ round libraries and confirmed the enrichment of sequences; then we read the sequences of the 12^th^ round library using a next-generation sequencer. Approximately 80% of ~1 million read sequences were covered by the six most frequently observed sequences (Table S1, Fig. 2A). We transcriptionally synthesised those six sequences for the following binding assay.

We selected the RNA sequence with the highest affinity for Ru(bpy)_3_^2+^ by titration, where changes in Ru(bpy)_3_^2+^ phosphorescence occurred with respect to concentrations of each RNA sequence (Fig. 2B). Among the six RNA sequences, the 12Rd-5 sequence exhibited the highest affinity for Ru(bpy)_3_^2+^ (*K*_d_ = 118 ± 90 nM). Here, we noted that RNA binding to Ru(bpy)_3_^2+^ suppressed non-radiative decays, leading to increases in phosphorescence intensity (discussed later). Interestingly, 12Rd-5 was not well populated, at only 1.5% after the 12 rounds of selection (Table S1, Fig. 2A). Although 12Rd-1 and 12Rd-2 were well populated (30.7% and 28.4%, respectively), both had only moderate affinities for Ru(bpy)_3_^2+^ (*K*_d_ = 1.6 and 1.0 μM, respectively). This was presumably because once such moderate-affinity sequences are well populated, it might be difficult to completely eliminate the sequences, even if one uses more stringent selection conditions during the subsequent rounds of selection^12^. We also note the possibility that 12Rd-3 and 12Rd-4, which did not exhibit binding to [Ru(bpy)_3_]^2+^, might have been selected due to their non-specific binding to the magnetic beads.

Another titration experiment using a series of partially truncated 12Rd-5 sequences clearly indicated the minimally required sequence for binding. The publicly available software Mfold^19^ predicted only one three-forked secondary structure for the 12Rd-5 sequence (Fig. S1). Given that such three-forked structures were reported as cocaine-binding aptamers^20^, we prepared seven RNA sequences lacking loops, stems, and/or hairpins (Table 1). The titration indicated that the 25 nucleotides at the 3′ end and the 5 nucleotides at the 5′ end were not required for binding (Fig. 2C). We designated this minimum-functional 53-nucleotide sequence as the Ru(bpy)_3_^2+^ aptamer (Fig. 2D). Although we explored several mutant variations of this aptamer that had the same predicted structure, we did not obtain a sequence that exhibited a significantly better affinity relative to the original aptamer sequence (Fig. S2).

### Enantioselective binding of the aptamer

**Figure 3:**
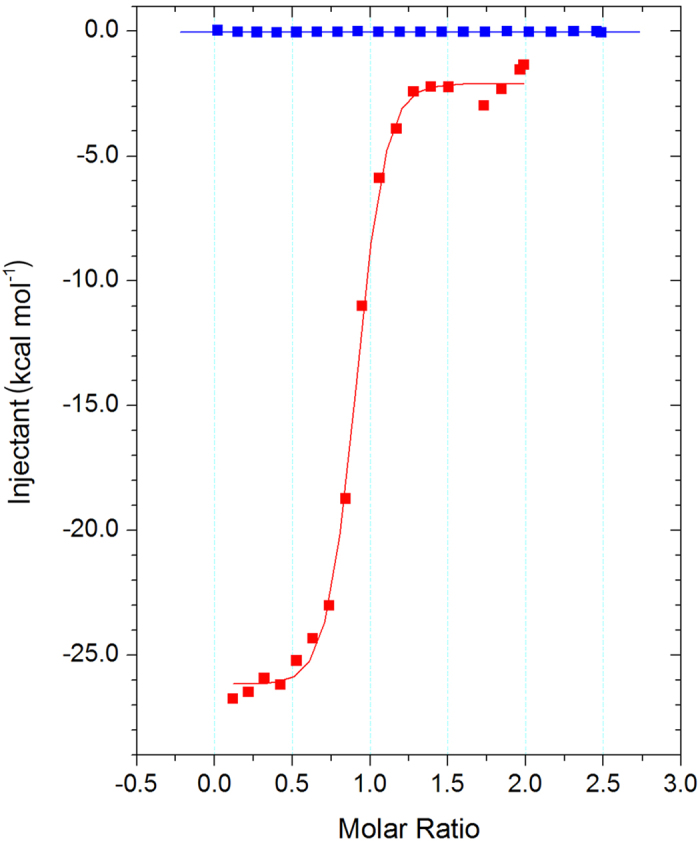
Specific binding of the aptamer to Λ-Ru(bpy)_3_^2+^. Isothermal titration calorimetry measurement clearly indicated that the aptamer strongly and specifically binds to Λ-Ru(bpy)_3_^2+^ (red, *K*_d_ = 65 nM), but not to Δ-Ru(bpy)_3_^2+^ (blue, *K*_d_ not determined). The determined thermodynamic parameters (Δ*H* = −27 kcal/mol and −TΔ*S* = +17 kcal/mol) implied that the aptamer changes its conformation upon Λ-Ru(bpy)_3_^2+^ binding.

Isothermal titration calorimetry (ITC) measurement using the two Ru(bpy)_3_^2+^ enantiomers demonstrated the enantioselectivity of this aptamer. The aptamer bound one of the enantiomers, Λ-Ru(bpy)_3_^2+^, but not the other, Δ-Ru(bpy)_3_^2+^ (Λ-Ru(bpy)_3_^2+^ *K*_d_ = 65 nM; binding was not detected for Δ-Ru(bpy)_3_^2+^; Fig. 3). This clear enantioselectivity suggested that the RNA binding is not trivial behaviour arising exclusively from the charged interactions between the positively charged Ru(bpy)_3_^2+^ and the negatively charged RNA aptamer. Notably, although there were some reports regarding the binding of a ruthenium complex to an oligonucleotide, the affinities were significantly lower than that determined in this study. For example, Satyanarayana *et al*.^21^ reported that a ruthenium phenanthroline complex, Ru(phen)_3_^2+^, binds dsDNA with *K*_d_ = 0.1 mM. Ruba *et al*.^22^ reported that, even when they used an intercalating ruthenium complex possessing one chelating ligand with a large aromatic structure, the affinity was sub-micromolar (*K*_d_ = 0.25 μM). Therefore, the nanomolar-scale affinity observed in this study is non-trivial, and we believe that such a high affinity could be attributed to the specific RNA sequence.

The thermodynamic parameters determined by ITC measurement (Δ*H* = −27 kcal/mol and −TΔ*S* = +17 kcal/mol) indicated that the strong binding was driven by enthalpy overcoming relatively large entropic loss. Enthalpy-driven binding with a loss of entropy was reported in a system involving RNA-conformational changes when the RNA binds a small compound. For example, Gilbert *et al*.^23^ reported such enthalpy-driven binding for 2,6-diaminopurine accompanied by RNA-conformational changes (Δ*H* = −40.3 kcal/mol and −TΔ*S* = +29.6 kcal/mol). However, Horowitz *et al*.^24^ reported increased entropy associated with the intercalation of proflavine to an oligonucleotide (Δ*H* = −2.6 kcal/mol and −TΔ*S* = −3.3 kcal/mol). Based on these comparisons, the aptamer presumably changes its conformation upon enthalpy-driven binding, with results from circular dichroism (CD) experiments supporting such aptamer-conformational changes (Fig. S3). The shape of the CD spectrum associated with the aptamer bound with Λ-Ru(bpy)_3_^2+^ was different from the simple sum of the individual CD spectra associated with the aptamer and Λ-Ru(bpy)_3_^2+^ separately. Furthermore, a cooperative melting curve was observed only for the aptamer with Λ-Ru(bpy)_3_^2+^, but not for the aptamer alone (Fig. S3B), suggesting that the aptamer folds into a specific structure when it binds Λ-Ru(bpy)_3_^2+^. Here, we also noted that the UV-Vis spectrum of Λ-Ru(bpy)_3_^2+^ was not affected by aptamer binding (data not shown), indicating that aptamer binding did not cause drastic Λ-Ru(bpy)_3_^2+^ deformation or decomposition. These findings indicated that the aptamer changed its conformation to achieve enthalpy-driven, high-affinity binding without Λ-Ru(bpy)_3_^2+^ deformation.

### Aptamer modulation of Ru(bpy)_3_^2+^ chemistry

**Figure 4:**
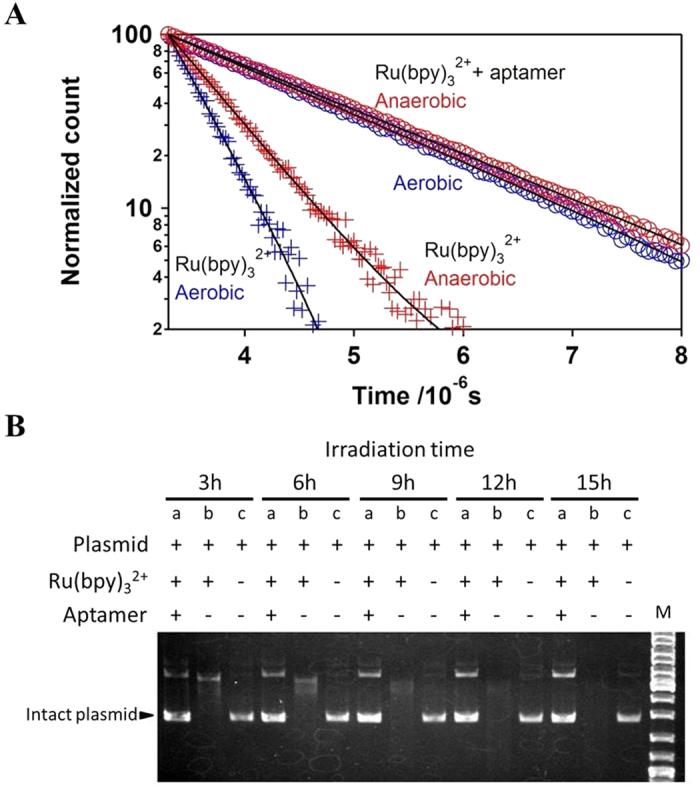
Aptamer binding elongates Λ-Ru(bpy)_3_^2+^ lifetime and suppresses O_2_ quenching. (**A**) Aptamer binding elongated the lifetime of ^3^MLCT state of Λ-Ru(bpy)_3_^2+^ from 380 ns (blue cross) to 1.66 μs (blue circle) under aerobic conditions. This was achieved by the suppression of non-radiative decay processes. The decay rate in the presence of the aptamer did not effectively depend upon O_2_ concentration, while the decay in the absence of the aptamer was dependent upon O_2_ concentration [red cross, Λ-Ru(bpy)_3_^2+^; red circles, Λ-Ru(bpy)_3_^2+^ with aptamer]. (**B**) Photocleavage-assay results indicated that aptamer binding suppressed O_2_ quenching. The plasmid alone (lane c) was intact, even after 15 h of irradiation. In the presence of Ru(bpy)_3_^2+^ (lane b), the plasmid was gradually and completely decomposed during irradiation. In the presence of both Ru(bpy)_3_^2+^ and the aptamer (lane a), the majority of the plasmid was still intact, even after 15 h of irradiation. Since the plasmid is photocleaved by the ^1^O_2_ produced by O_2_ quenching of the ^3^MLCT state, these results indicated that aptamer binding suppressed O_2_ quenching.

The increased phosphorescence intensity correlated with an elongated ^3^MLCT-state lifetime. Aptamer binding markedly elongated the lifetime by four-fold to 1.65 μs under aerobic conditions (blue crosses and circles in Fig. 4A; Table 2). The elongated lifetime was substantially longer than the reported lifetimes observed in various organic solvents under anaerobic conditions (765 ns in methanol^7^, 855 ns in acetonitrile^25^, and 938 ns in propylene carbonate^25^) and in micelles^26^, indicating that the aptamer furnished a distinctive environment for the ^3^MLCT state. Such a distinctive environment was supported by the blue-shift of the emission peak from 627 nm to 598 nm associated with aptamer binding (Fig. S4). Because the peak wavelength of the aptamer-bound Λ-Ru(bpy)_3_^2+^ was shorter than that of tris(2,2′-bipyridine)ruthenium(II) hexafluorophosphate [Ru(bpy)_3_(PF_6_)_2_] in dichloromethane (606 nm), the dielectric constant (*ε*) of the aptamer was deduced to be smaller than that of dichloromethane (*ε* = 8.93). Such a low dielectric constant in the aptamer would contribute to the elongated ^3^MLCT-state lifetime.

The lifetime of an excited state is defined as the average time that the molecule spends in the excited state prior to returning to the ground state through radiative and non-radiative decays^27^:


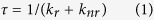


where *τ* is the lifetime of the ^3^MLCT state, *k*_r_ is a radiative rate, and *k*_nr_ is the sum of the decay rates associated with all possible types of non-radiative decays. Additionally, both *k*_r_ and *k*_nr_ are highly correlated with quantum yield (*Q*_*Y*_), which is defined by the fraction that exhibits decay through radiation^27^:


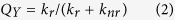


The radiative- and non-radiative-decay rates determined using Eqs 1 and 2 clearly indicated that aptamer binding barely decreased the rate of radiative decay from 1.00 × 10^5^ s^−1^ to 0.87 × 10^5^ s^−1^, whereas it substantially decreased the rate of non-radiative decay from 25.3 × 10^5^ s^−1^ to 5.19 × 10^5^ s^−1^ (Table 2). Therefore, the ^3^MLCT-state lifetime elongation and the increased phosphorescence intensity predominantly arose from the suppression of non-radiative decay.

One type of non-radiative decays that occurs under aerobic conditions is O_2_ quenching. We determined the O_2_ bimolecular-quenching constant (*k*_*q*_^O2^) using a pseudo-first-order linear relationship^28^:


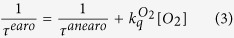


where the two superscripts on *τ, aero* and *anaero*, denote the ^3^MLCT-state lifetime under aerobic and anaerobic conditions, respectively, and [O_2_] denotes the oxygen concentration (in this study, we used 0.26 mM O_2_ in equilibrated H_2_O at 25 °C). Aptamer binding markedly decreased *k*_*q*_^O2^ from 3.5 × 10^9^ M^−1^ s^−1^ to 0.13 × 10^9^ M^−1^ s^−1^, indicating that O_2_ quenching was effectively suppressed by the aptamer. To experimentally evaluate the suppression of O_2_ quenching, we examined the photocleavage of a plasmid by ^1^O_2_ produced via O_2_ quenching. Irradiation of a solution containing Λ-Ru(bpy)_3_^2+^ and a plasmid with visible light resulted in nearly complete degradation of the plasmid; however, such photodegradation was effectively suppressed by the addition of the aptamer (Fig. 4B). Given that the reduction potential of the ^3^MLCT state is unfavourable for driving guanine oxidation^29^, DNA is degraded by ^1^O_2_ produced via O_2_ quenching^30^. The suppression of photodegradation by aptamer binding clearly indicated that aptamer binding substantially suppressed O_2_ quenching.

While the aptamer suppressed O_2_ quenching, it did not disrupt quenching by another source. Specifically, we employed a quencher, *N,N*′-dimethyl-4,4′-bipyridinium dichloride [methyl viologen (MV)], a known quencher of the ^3^MLCT state via charge transfer at nearly diffusion limits^31^,^32^. We determined the bimolecular-quenching constant of the quencher (*k*_q_^Q^) using the Stern–Volmer equation^7^,^27^,^32^:


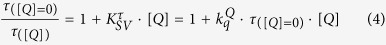


where *K*_sv_^*τ*^ denotes the Stern–Volmer constant and *τ*_([Q]=0)_ and *τ*_([Q])_ denote the lifetime in the absence and presence of a certain concentration of a quencher ([*Q*]), respectively. The determined *k*_q_^MV^ values in the presence and absence of the aptamer were effectively the same at 1.94 × 10^9^ s^−1^ and 1.69 × 10^9^ s^−1^ (Table 3), respectively, indicating that the aptamer did not interfere with the charge transfer from the ^3^MLCT state to MV.

The discrepancy between O_2_ quenching and MV quenching presumably arises due to the differences in their quenching mechanisms. Extensive studies on Ru(bpy)_3_^2+^ and MV demonstrated that MV quenching arises due to the charge transfer from the ^3^MLCT state to MV^27^,^31^. Because the back-electron-transfer rate from MV to Ru(bpy)_3_^3+^ within the solvent cage is too fast, the charge transfer does not effectively yield redox products. For O_2_ quenching, Mulazzani *et al*.^28^ reported that the ^3^MLCT state was quenched by both charge- and energy-transfer mechanisms, with equal bimolecular quenching-rate constants^28^. Similar to the charge transfer observed for MV quenching, that for O_2_ quenching also does not effectively yield redox products. In contrast to the charge transfer, the energy transfer to O_2_ produces ^1^O_2_, which acts as a strong oxidant. Because electronic interactions between the excited Ru(bpy)_3_^2+^ and O_2_ within a collision complex are required for the energy transfer, it might be reasonable to presume that the 17-kDa aptamer disrupts such close contacts to cause overlap of the electron cloud between 600-Da Ru(bpy)_3_^2+^ and O_2_. The size of the aptamer is similar to those of small proteins with cofactors (e.g., myoglobin and cytochrome *c*), implying that this size might be required for specific binding to a cofactor. In addition to binding to Ru(bpy)_3_^2+^, our aptamer strengthened the key property required for attractive photochemical reactions. Therefore, we successfully demonstrated the artificial evolution of a biomolecule to regulate the chemistry of a fifth-period metal.

Here, we reported the selection of an RNA aptamer that binds Ru(bpy)_3_^2+^. The newly discovered aptamer strongly (*K*_d_ = 65 nM) and enantioselectively bound Λ-Ru(bpy)_3_^2+^, selectively suppressed the deactivation process via energy transfer, and elongated the lifetime of its photo-excited state by four-fold. Although aptamer binding suppressed energy transfer, it did not disturb charge transfer, indicating that the aptamer presented in this study allocated the energy of the photo-excited state to charge transfer. The charge transfer of Ru(bpy)_3_^2+^ to MV has attracted extensive attention due to the intriguing photoredox possibilities associated with its capacity to split water into H_2_ and O_2_ through the use of visible light. To effectively process such chemical reactions, energy allocation could play an important role. Besides, an *in vivo* observation system that harnesses the properties of Ru(bpy)_3_^2+ ^18,33 could be improved by employing the aptamer to modulate those properties. We believe that our successful demonstration of selective-energy allocation by an RNA aptamer illustrates a broader point concerning the potential power of combining *in vitro*-created biomolecules with non-biological reactants to perform enhanced chemical reactions.

## Methods

### Selection of the RNA aptamer

Selection of the RNA aptamer was performed according to Nakamura and coworkers^34^, with some modifications. All DNA templates and primers were purchased from Eurofins Genomics (Kawasaki, Japan). We prepared a dsDNA library by synthesising the complementary strands of an ssDNA library (5′-CTCTCATGTCGGCCGTTA-N_50_-CGTCCATTGTGTCCCTATAGTGAGTCGTATTA-3′) using a forward primer (5′-TAATACGACTCACTATAGGGACACAATGGACG-3′; the underlined sequence is the T7 promoter) and PrimeSTAR^®^ GXL DNA polymerase (Takara Bio, Shiga, Japan). The dsDNA library was transcribed into an RNA library using the MEGAscript^®^ T7 transcription kit (Life Technologies, Carlsbad, CA, USA). Because the transcription starts from the three guanine nucleotides (GGG) located just after the T7 promoter, the RNA sequences that we used in this study were 83 nucleotides in length. After purification with an RNA Clean & Concentrator™ kit (Zymo Research, Irvine, CA, USA) and refolding [85 °C for 10 min, followed by cooling at 4 °C in a thermal cycler (S1000; Bio-Rad, Hercules, CA, USA)], the RNA library was incubated with magnetic beads modified with a derivative of Ru(bpy)_3_^2+^ for 1 h at 4 °C in 4-(2-hydroxyethyl)-1-piperazineethanesulfonic acid (HEPES) buffer [20 mM HEPES, 100 mM NaCl, and 5 mM MgCl_2_ (pH 7.0)]. We used 1.8 × 10^15^ molecules of RNA for the first and second rounds of selection and 4 × 10^14^ molecules for the later rounds of selection. Magnetic beads coated with amine groups (Dynabeads^®^ M-270 Amine; Life Technologies) were covalently coupled with the *N*-hydroxysulfosuccinimide-activated Ru(bpy)_3_^2+^ derivative [*bis*(2,2′-bipyridine)-4′-methyl-4-carboxybipyridine-ruthenium *N*-succinimidylester-*bis*(hexafluorophosphate); Sigma-Aldrich, St. Louis, MO, USA]. We used 5 × 10^6^ modified beads for each round of selection. Unbound RNA was washed eight times with the HEPES buffer. The bound RNA was eluted with an elution buffer [(7 M urea, 20 mM HEPES, 100 mM NaCl, and 5 mM MgCl_2_ (pH 7.0)] for the 1^st^ and 2^nd^ rounds of elutions, then 10 mM Ru(bpy)_3_Cl_2_ was added to the HEPES buffer for rounds three through eight, 10 μM Ru(bpy)_3_Cl_2_ for rounds nine and 10, and 10 nM Ru(bpy)_3_Cl_2_ for rounds 11 and 12 [Ru(bpy)_3_Cl_2_; Tris(2,2′-bipyridyl)dichlororuthenium(II) hexahydrate; Sigma-Aldrich]. After incubation in the elution buffer for 30 min at 4 °C, the solution was collected and filtered using an empty column (Micro Bio-Spin^®^ Columns; Bio-Rad) to eliminate the magnetic beads. Eluted RNA was purified and subjected to reverse transcription (PrimeScript™ RT-PCR kit; Takara Bio) with a reverse primer (5′-CTCTCATGTCGGCCGTTA-3′). After PCR using the previously indicated forward and reverse primers, the obtained dsDNA was used for the next selection round. The DNA sequences were read after the 12th round using a next-generation sequencer (MiSeq; Takara Bio). We obtained 2,490,324 total reads and then abstracted 1,181,011 reads by filtering with the exact length in between the primer sequences. We clustered identical sequences in the filtered reads using CD-HIT^35^ (Table. S1). We determined the concentration of Ru(bpy)_3_Cl_2_ using the extinction coefficient of 14,600 at 452 nm^1^.

### Phosphorescence measurements

We transcriptionally synthesised all RNA sequences [the sizes of the RNA products were confirmed by gel electrophoresis and matrix-assisted laser desorption/ionisation time-of-flight mass spectrometry (Microflex; Bruker Corporation, Billerica, MA, USA)], then prepared a solution containing 1 μM Ru(bpy)_3_Cl_2_ and RNA at a series of different concentrations (from 1 to 10 μM) in HEPES buffer. *K*_d_ values were calculated using Igor Pro (WaveMetrics, Lake Oswego, OR, USA). RNA concentrations were determined using extinction coefficients estimated by the nearest-neighbour method (540,700 M^−1^ cm^−1^ for the aptamer). Emission spectra were obtained by blue-light excitation at 470 ± 10 nm using a NanoDrop™ 3300 fluorospectrometer (Thermo Scientific, Waltham, MA, USA). Emission intensities at 595 nm were normalised with the intensity of 1 μM Ru(bpy)_3_^2+^ solution without RNA. The emission peak positions were determined using a fluorimeter equipped with a light source for wavelength calibration (FP-8500 and ESC-842; JASCO, Tokyo, Japan). We also synthesised the aptamer with 2′-F RNA using a DuraScribe^®^ T7 Transcription Kit (Epicentre, Madison, WI, USA), but the 2′-F aptamer did not exhibit binding to [Ru(bpy)_3_]^2+^.

### ITC

We dialysed the RNA aptamer overnight at 4 °C in phosphate-buffered saline (T900; Takara Bio) and then allowed the RNA to refold as described above in “Selection of the RNA aptamer”. *Δ*- and *Λ*-Ru(bpy)_3_Cl_2_ were dissolved in the same buffer used for aptamer dialysis. We performed ITC (MicroCal iTC_200_; GE Healthcare Life Sciences, Pittsburgh, PA, USA) with 5 μM aptamer in the ITC cell and 50 μM Ru(bpy)_3_Cl_2_ in the ITC syringe. Data were analysed with one set of site-fitting modes (MicroCal iTC_200_ Data Analysis software; GE Healthcare Life Sciences).

### Lifetime measurement

Phosphorescence lifetime was determined using a time-correlated single-photon counting (TCSPC) system (DCS-120 system; Becker and Hickl GmbH, Berlin, Germany). The excitation wavelength was 473 nm, and the emission was collected using a long-pass filter (>495 nm). The lifetime measurement was conducted at room temperature at a solution temperature of 26 ± 1 °C. To saturate aptamer binding, we used 4 μM aptamer and 1 μM *Λ*-Ru(bpy)_3_Cl_2_ in HEPES buffer. The anaerobic samples were prepared using the freeze-pump-thaw method, and we mixed the solutions in a globe box (G-10N-MV-AV; Takasugi-Seisakusyo, Tokyo, Japan) as needed.

### Estimation of quantum yield

First, we determined the absolute quantum yields of *Λ*-Ru(bpy)_3_^2+^ and aptamer-bound *Λ*-Ru(bpy)_3_^2+^ under aerobic conditions. Next, we determined the relative quantum yields of *Λ*-Ru(bpy)_3_^2+^ and aptamer-bound *Λ*-Ru(bpy)_3_^2+^ under aerobic conditions from their respective absolute quantum yields according to a previously reported method^36^. The anaerobic samples were prepared by three cycles of freeze-pump-thaw, and absorbance and fluorescence spectra were recorded using a UV-Vis-absorption spectrometer (JASCO) and a fluorimeter (F-6500; JASCO). Measurements were conducted at 25 ± 1 °C.

### Stern–Volmer plot

The bimolecular-quenching constant was estimated using a Stern–Volmer plot. We prepared sample solutions of 4 μM aptamer, 1 μM *Λ*-Ru(bpy)_3_^2+^, and a series of concentrations of *N,N*’-dimethyl-4,4′-bipyridinium dichloride (Wako Pure Chemical Industries). After quencher addition, we measured phosphorescence using a NanoDrop™ 3300 fluorospectrometer (Thermo Scientific), and lifetime was determined using the TCSPC system (Becker and Hickl GmbH).

### Photocleavage assay

We prepared a solution containing 70 ng/mL plasmid (pTolA3b, a derivative of pUC) with or without the other two components [30 μM *Λ*-Ru(bpy)_3_^2+^ and 75 μM aptamer (final concentrations)]. We irradiated the solutions using white light-emitting diodes (LC-LED 450 W; Titec, Tokyo, Japan) at their maximum output (165 μM flux density). After irradiation (up to 15 h) we ran gel electrophoresis using an E-Gel^®^ system (Life Technologies).

## Additional Information

**How to cite this article**: Thoa, T. T. T. *et al*. Regulation of photosensitisation processes by an RNA aptamer. *Sci. Rep.* **7**, 43272; doi: 10.1038/srep43272 (2017).

**Publisher's note:** Springer Nature remains neutral with regard to jurisdictional claims in published maps and institutional affiliations.

## Supplementary Material

Supplementary Information

## Figures and Tables

**Figure 1:**
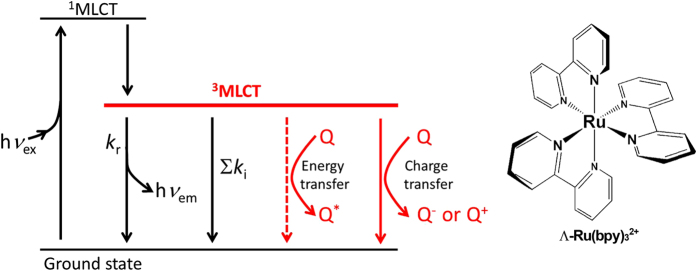
The deactivation processes of the photo-excited triplet state of Ru(bpy)_3_^2+^. This represents a long-lived triplet state produced by ultrafast intersystem crossing from the excited singlet state that is deactivated via multiple mechanisms, including radiative decay (*k*_r_), and non-radiative mechanisms, including energy transfer, charge transfer, and others (denoted by ∑*k*_i_). Here, we show that the RNA aptamer presented in this study binds Ru(bpy)_3_^2+^ and suppresses energy transfer (broken lines), leading to a longer excited-state lifetime and enhanced photo-reactivity.

**Table 1:**
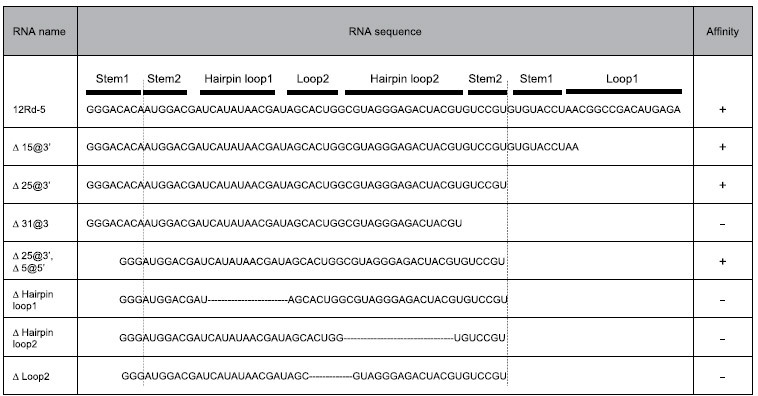
Truncated RNA sequences used to clarify the minimum sequence required for binding.

Note that we maintained the GGG sequence at the 3′ end to encourage effective transcription.

**Table 2:** Spectroscopic parameters.

		*Q*_*Y*_^a^	*τ*/μs^b^	*k*_r_/10^5^ s^−1^	*k*_nr_/10^5^ s^−1^
Aerobic	Ru(bpy)_3_^2+^	0.038	0.38	1.00	25.32
	Ru(bpy)_3_^2^ + aptamer	0.144	1.65	0.87	5.19
Anaerobic	Ru(bpy)_3_^2+^	0.064	0.58	1.10	16.14
	Ru(bpy)_3_^2^ + aptamer	0.155	1.75	0.89	4.83

^a^The relative quantum yield determined using the absolute quantum yield of Ru(bpy)_3_^2+^ under aerobic conditions. We estimated 10% and 40% errors for the absolute and relative quantum yields, respectively.

^b^Standard deviation: ~2%.

**Table 3:** The parameters determined using the Stern-Volmer equation.

		*K*_*SV*_^*I*^/10^2^ M^−1^^a^	*K*_*SV*_^*τ*^/10^2^ M^−1^^b^	*k*_q_^MV^/10^9^ M^−1^ s^−1^^c^
Aerobic	Ru(bpy)_3_^2+^	7.46	5.25	1.69
	Ru(bpy)_3_^2+^ + aptamer	29.4	33.4	1.94
Anaerobic	Ru(bpy)_3_^2+^	7.16	7.66	1.30
	Ru(bpy)_3_^2+^ + aptamer	20.3	34.5	1.35

^a^95% confidence interval: ~4% to 12%.

^b^95% confidence interval: ~3% to 12%.

^c^95% confidence interval: ~3% to 8%.
