# Supplementary material for: Regulation of photosensitisation processes by an RNA aptamer
Source: Sci Rep. 2017 Feb 24;7:43272. doi: 10.1038/srep43272 (PMC5324105; doi:10.1038/srep43272)
Supplement: Supplementary Information [file srep43272-s1.pdf]

Regulation of photosensitisation processes by an RNA aptamer

Tran Thi Thanh Thoa, Noriko Minagawa, Toshiro Aigaki, Yoshihiro Ito, Takanori Uzawa

Supplementary Methods

Chiral separation

We separated $Ru(bpy)_3Cl_2$ enantiomers using a chiral separation column. $Ru(bpy)_3Cl_2$ (10 mg/mL) was dissolved in running solution [80% acetonitrile, 20% H_2O, and 0.02% trifluoroacetic acid (TFA)]. A high-performance liquid chromatograph (JASCO, Tokyo, Japan) equipped with a CHIRALPAK® IA column (20 mm × 250 mm; Daicel, Osaka, Japan) was employed at a flow rate of 3 mL/min to separate the enantiomers. To exchange the counter ion with Cl^-, the eluate from the chiral column was subjected to anion-exchange chromatography (DOWEX 1 × 8; Wako Pure Chemical Industries, Osaka, Japan). Both enantiomers were confirmed by matrix-assisted laser desorption/ionisation time-of-flight mass spectrometry (Microflex; Bruker Daltonics, Raleigh, NC, USA) and circular dichroism using a spectropolarimeter (J-720; JASCO). The purity of the separated enantiomers was analysed using an OZ-H column (4.6 mm × 250 mm; Daicel) with diethanolamine solution (100 acetonitrile : 0.1 TFA : 0.1 diethylamine) at 1 mL/min. We used enantiomers exhibiting > 95% purity for all experiments.

Supplementary Data

Table S1. The six most frequently (> 1.0 %) observed sequences.

	RNA sequence	Population	
		Frequency /1,181,011	%
12Rd-1	5′-GGGACACAAUGGACGGUACGUAUUGCGUGGGACCCCGCGCAAUGAACUGGCCGAGGGAGACUCGG UAACGGCCGACAUGAGAG-3′	362,442	30.7
12Rd-2	5′-GGGACACAAUGGACGGCAGAUCUGAGAGGGGAAGACCCUUUGUUAUUAACGAACUUGCGGUAUGA UAACGGCCGACAUGAGAG-3′	335,710	28.4
12Rd-3	5′-GGGACACAAUGGACGUGGGGCUACAGACCAUCCGUUCUAUCCCGAGCACCGGGCCAAUCUCAAGU UAACGGCCGACAUGAGAG-3′	147,880	12.5
12Rd-4	5′-GGGACACAAUGGACGGGCACAAGGUCCCACCAUCUCAGACGAGAAAGGAUACGCAGAUCGGCGAA UAACGGCCGACAUGAGAG-3′	68,510	5.8
12Rd-5	5′-GGGACACAAUGGACGAUCAUAUAACGAUAGCACUGGCGUAGGGAGACUACGUGUCCGUGUGUACC UAACGGCCGACAUGAGAG-3′	17,315	1.5
12Rd-6	5′-GGGACACAAUGGACGCGGGACUUAAUGCUGCUCAAGGGUGCGAUGAACUAAGAAAAUCGCCCGCG UAACGGCCGACAUGAGAG-3′	11,847	1.0
Others		237,307	20.1

Note that the above sequences contain the fixed regions at the 5′ and 3′ termini. The library we used was 5′-TAATACGACTCACTATAGGGACACAATGGACG-**N**$_{50}$-TAACGGCCGACATGAGAG-3′.

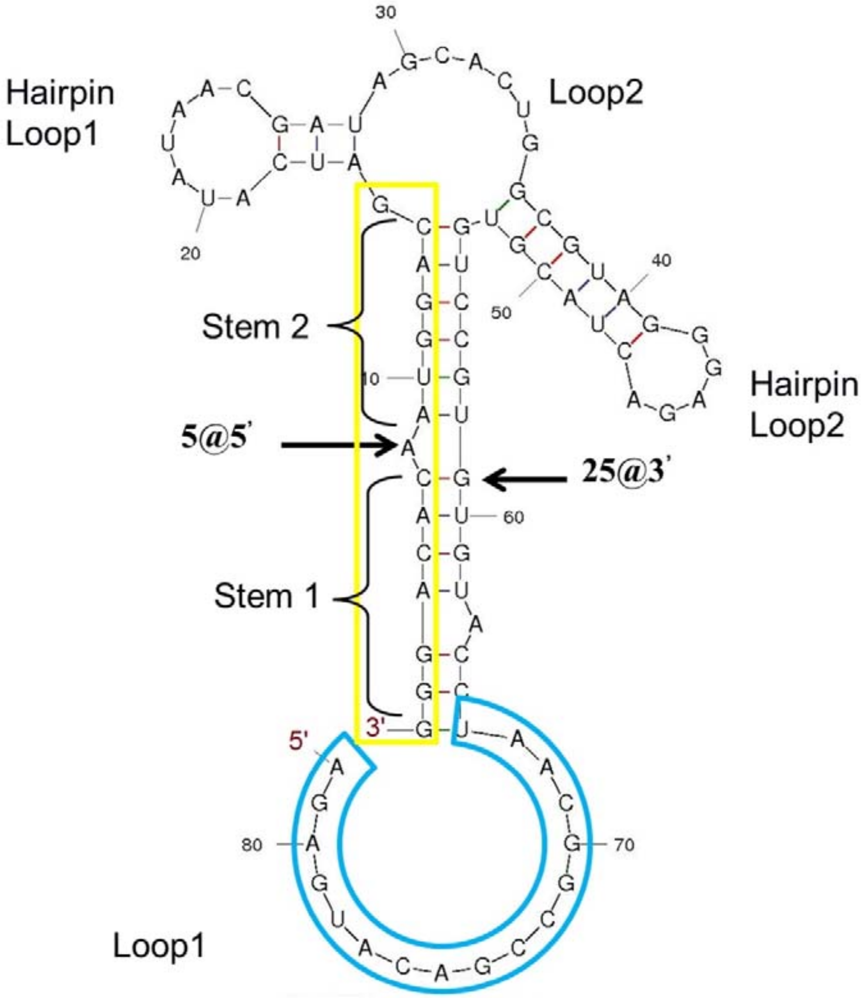

Fig. S1. Predicted secondary structure. The three-forked secondary structure predicted for the 12Rd-5 sequence. The fixed regions at the 5′ and 3′ termini are circles with yellow and light blue lines.

Mutant aptamers with the same secondary structure as the native aptamer

A

Original	GGGAUGGACGAUCAUAUAACGAUAGCACUGGCGUAGGGAGACUACGUGUCCGU
Mut1	GGGAGUGACGAUCAUAUAACGAUAGCACUGGAGUCGGGAGACGACUUGUCACU
Mut2	GGGUUGGUCGAUCAUAUAACGAUAGCACUGCCUAUAGGAGAUAUAGGGACCAA
Mut3	GGGUACGGCGAUCAUAUAACGAUAGCACUGUCUAUAGGAGAUAUAGAGCCGUA
Mut4	GGGAGUGACGAUCAUAUAACGAUAGCACUGUCUAUAGGAGAUAUAGAGUCACU
Mut5	GGGUUGGUCGAUCAUAUAACGAUAGCACUGGAGUCGGGAGACGACUUGACCAA

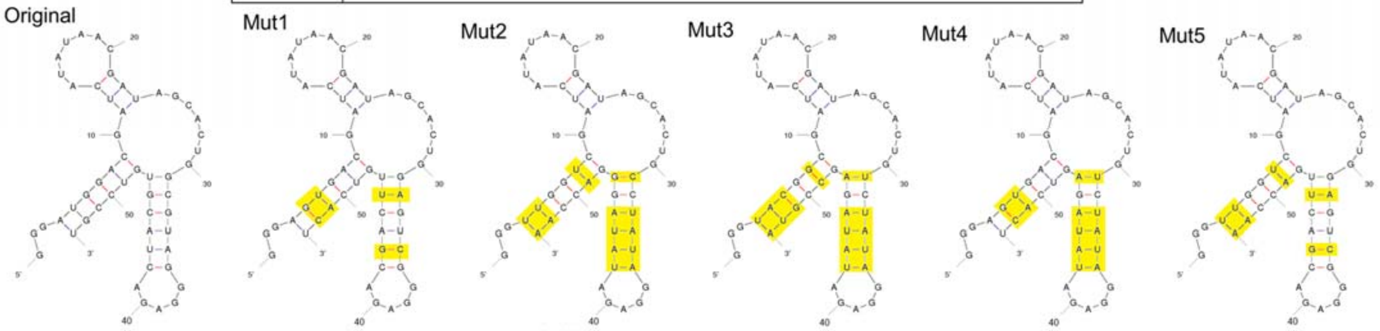

B

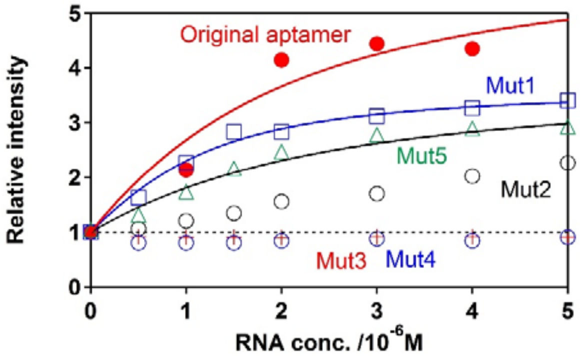

Fig. S2. Mutations of the aptamer indicate that the modulation of the ^{3}MLCT state of $Ru(bpy)_3^{2+}$ is not trivial. Although we explored several mutant variations of this aptamer with the same predicted structure, we did not obtain a sequence that exhibited a significantly better affinity relative to the original aptamer sequence.

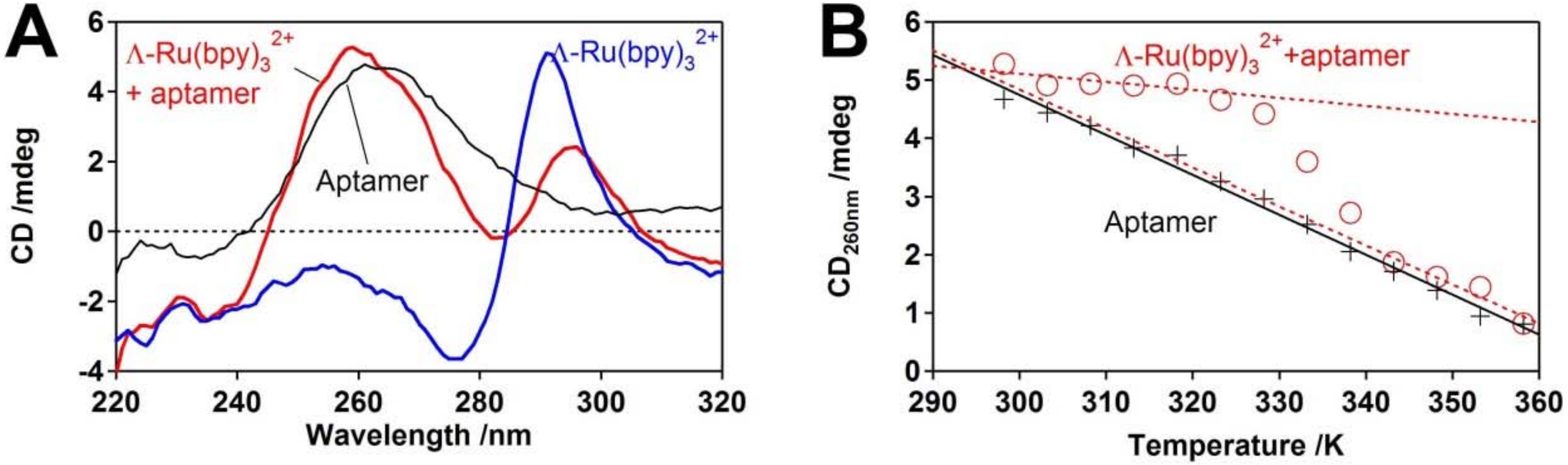

Fig. S3. Conformational changes in the aptamer following Λ-Ru(bpy)$_3$$^{2+}$ binding. Circular dichroism (CD) results indicate a conformational change in the aptamer upon binding to Λ-Ru(bpy)$_3$$^{2+}$. (A) The shape of the spectrum associated with the aptamer in the presence of Λ-Ru(bpy)$_3$$^{2+}$ (red) is not a simple sum of the individual CD spectra from the aptamer (black) and Λ-Ru(bpy)$_3$$^{2+}$ (blue). (B) A cooperative melting curve was observed for the aptamer in the presence of Λ-Ru(bpy)$_3$$^{2+}$, which not seen for the aptamer alone. The two red-coloured dotted lines allow visualisation of the cooperativity.

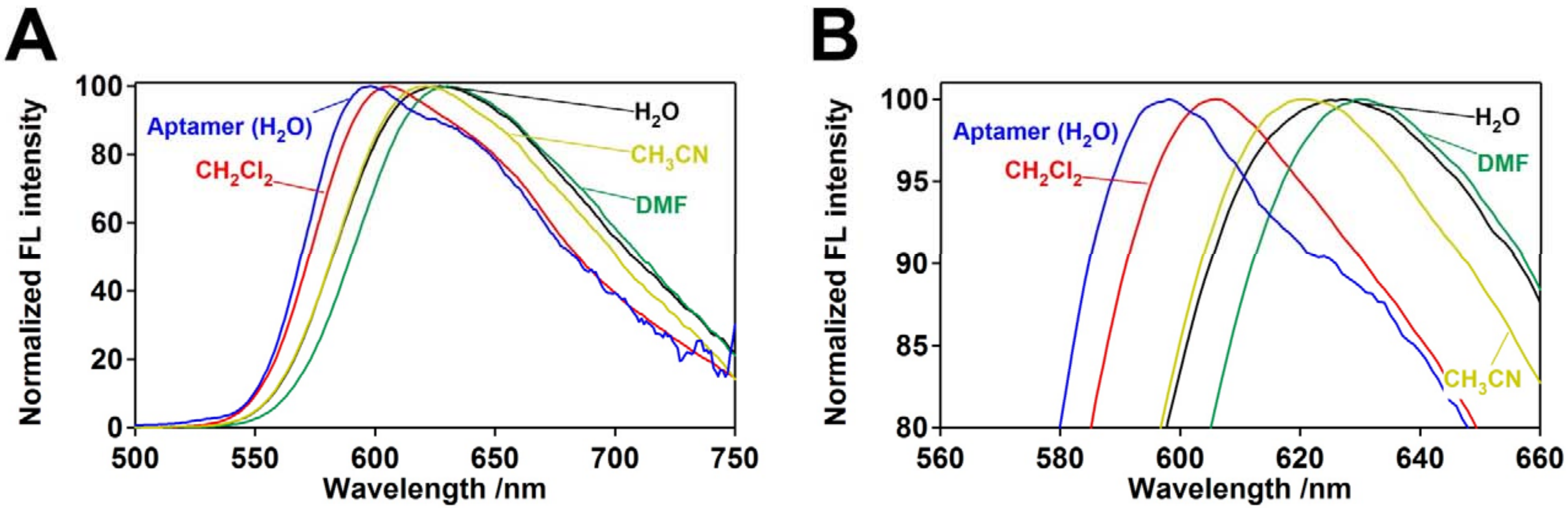

Fig. S4. The aptamer binding drastically blue-shifts the emission peak maximum of Λ-Ru(bpy)$_3^{2+}$. The dielectric constants of dichloromethane (CH_2Cl_2), acetonitrile (CH_3CN), *N*,*N*-dimethylformamide (DMF), and H_2O are 8.93, 37.5, 36.7, and 80.1, respectively. The peak wavelength of the aptamer-bound Λ-Ru(bpy)$_3^{2+}$ was found to be shorter than that of Ru(bpy)$_3^{2+}$ in dichloromethane. Therefore, the dielectric constant of the aptamer was deduced to be smaller than that of dichloromethane.
